# Current Sample Preparation Methods and Determination Techniques for the Determination of Phthalic Acid Ester Plasticizers in Edible Oils

**DOI:** 10.3390/molecules28135106

**Published:** 2023-06-29

**Authors:** Menghui Qi, Yanyan Li, Zheng Zhu, Bin Du, Di Chen

**Affiliations:** 1 School of Pharmaceutical Sciences, Zhengzhou University, Zhengzhou 450001, China; 13607692653@163.com (M.Q.); liyanyan2024@163.com (Y.L.); zhuzheng@zzu.edu.cn (Z.Z.); 2 Key Laboratory of Targeting Therapy and Diagnosis for Critical Diseases of Henan Province, Zhengzhou 450001, China

**Keywords:** edible oils, phthalic acid esters, sample preparation methods, detection techniques

## Abstract

In the process of production, processing, transportation, and storage of edible oils, the oils inevitably come into contact with plastic products. As a result, plasticizers migrate into edible oils, are harmful to human health, and can exhibit reproductive toxicity. Therefore, the determination of plasticizers in edible oils is very important, and a series of sample preparation methods and determination techniques have been developed for the determination of plasticizers in edible oils. Phthalic acid ester (PAE) plasticizers are the most widely used among all plasticizers. This review aims to provide a comprehensive overview of the sample preparation methods and detection techniques reported for the determination of PAEs in edible oils since 2010, focusing on sample preparation methods of edible oils combined with various separation-based analytical techniques, such as gas chromatography (GC) and liquid chromatography (LC) with different detectors. Furthermore, the advantages, disadvantages, and limitations of these techniques as well as the prospective future developments are also discussed.

## 1. Introduction

Plasticizers are a common class of chemical additives in the manufacturing process of plastics. They are widely used in plastic products to improve the flexibility, extensibility, and durability of the product. A wide variety of plasticizers are available for commercialization in the food field. Phthalic acid esters (PAEs) are the most widely used among all the plasticizers [1]. The commonly used phthalates include dimethyl phthalate (DMP), diethyl phthalate (DEP), dibutyl phthalate (DBP), benzyl butyl phthalate (BBP), di-2-(ethylhexyl) phthalate (DEHP), dicyclohexyl phthalate (DCHP), and di-isononyl phthalate (DINP) [2]. The physical and chemical information of the above-mentioned phthalates are summarized in Table 1.

PAEs are widely used in the plastic packaging materials of foods and beverages. However, PAEs are prone to migrating from packaging into foods or beverages because PAEs are only physically bonded to the plastic polymer macromolecules by hydrogen bonding or van der Waals forces instead of close chemical bonding [3]. In particular, due to the lipophilicity and hydrophobicity of PAEs, edible oils are more likely to be polluted by PAEs. During the production and storage, edible oils inevitably come into contact with plastic products, such as machines, equipment, and containers. PAEs can easily migrate from plastic containers into oils [4]. Luo et al. indicated that the human daily intake of PAEs via edible oils is much higher than that via bottled water [5]. Although PAEs have fewer acute toxic and side effects, long-term intake of PAEs will have many adverse effects on human health, especially human reproduction [6,7], development [8,9], cardiovascular systems [10], and liver and kidneys [11,12]. The endocrine-disrupting toxicity [13], neurotoxicity [14], and carcinogenic toxicity [15] of PAEs have also been reported. Therefore, to guarantee oil quality and human health, it is necessary to accurately determine the contents of PAEs in edible oils.

**Figure 1:**
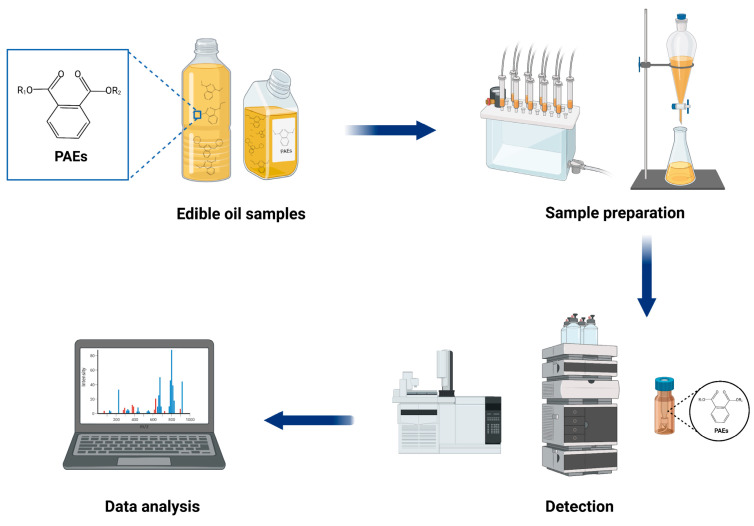
Schematic diagram of the typical analytical procedure for the detection of PAEs in edible oils. (Created with BioRender.com).

With the continuous improvement of people’s living standards and health awareness, the detection of PAEs in edible oils has attracted more and more attention, and various analytical methods have emerged. Given the complex matrix of edible oils and the strong lipophilicity and low concentration of PAEs, it is difficult to fully extract and accurately analyze the contents of PAEs in edible oils. Therefore, selecting efficient sample preparation methods and appropriate determination techniques is essential for the successful detection of PAEs in edible oils. With the development of modern science and technology, new sample preparation and analytical techniques for the determination of PAEs in edible oils have been developed continuously in recent years. Herein, we reviewed and summarized the advances in sample preparation methods and detection techniques for the determination of PAEs in edible oils from 2010 to present (Figure 1). This review is expected to provide ideas for researchers to establish more appropriate analytical methods.

## 2. Sample Preparation Methods

The matrix of edible oils is complex, and it is necessary to perform efficient sample preparation to extract the target components before detection [16,17,18]. Sample preparation generally includes steps such as sample collection, extraction, purification, and concentration. At present, the sample preparation methods for detecting PAEs in edible oils mainly include liquid-based extraction techniques [19,20,21,22], gel permeation chromatography (GPC) [23,24], sorptive-based extraction techniques [25,26,27], and QuEChERS (quick, easy, cheap, effective, rugged, and safe) [28,29,30].

### 2.1. Liquid-Based Extraction Techniques

Liquid-liquid extraction (LLE) is a traditional method for sample preparation. It transfers target analytes from the sample solution to the extractant based on the different distribution coefficients of the target analytes in two immiscible or slightly soluble solvents. For the extraction of PAEs from edible oils, the commonly used extraction solvents are mainly nonpolar solvents such as n-hexane, isooctane, and dichloromethane. Table 2 summarizes the methods for analyzing PAEs in edible oils by LLE combined with appropriate determination techniques.

LLE is simple to perform and does not require expensive instruments, making it widely used in the detection of PAEs [19,20,21,22]. However, traditional LLE is not environmentally friendly because it often results in the formation of emulsions and consumes large volumes of organic solvents during the extraction process. Zhou et al. used hexane-saturated acetonitrile (ACN) to extract PAEs from edible oils. The entire extraction process took more than 15 min, with a high consumption of over 40 mL of hexane-saturated acetonitrile and approximately 0.5 g of vegetable oils [22].

With the development of the new concept of green chemistry, the advantages of microextraction technology have become prominent [34]. Compared with traditional extraction methods, microextraction technology consumes lower amounts of organic solvents and is more environmentally friendly [35,36,37]. Liquid-phase microextraction (LPME) is a miniaturized extraction technology developed on the basis of LLE. Since the introduction of LPME in 1996 [38], this technology has been widely used in the analysis of various samples as a new approach for sample preparation [32,39,40]. In recent years, various modes have been derived from LPME, among which dispersed liquid–liquid microextraction (DLLME) has been widely used for the extraction of PAEs in edible oils. The DLLME system is a ternary solvent system composed of samples, dispersants, and extractants. During DLLME, a mixture of extractant and dispersant is quickly injected into the sample solution through a syringe. Under the action of the dispersant, the extractant that was originally immiscible with the sample solution forms small droplets. The droplets are dispersed in the extractant, thus increasing the contact area between the extraction phase and the sample, shortening the extraction time. Currently, these extraction technologies are often combined with auxiliary means, such as ultrasonic-assisted extraction [41] and air-assisted extraction [32,33,40,41], for the extraction of PAEs in edible oils. These auxiliary methods can further reduce the consumption of organic solvents and effectively improve the extraction efficiency. Khoshmaram et al. used air-assisted liquid–liquid extraction (AA-LLE) coupled with DLLME for the extraction and preconcentration of some PAEs from edible oils prior to their detection by gas chromatography (GC). The entire extraction process only required about 10 min, with the low consumption of 1 mL of dimethyl sulfoxide and 0.45 mL of chloroform as the extractant and 0.5 mL of edible oils [33].

Extraction technologies based on traditional LLE generally use organic solvents as extractants, which are harmful to the operator and the environment. Farajzadeh et al. used an alkaline aqueous solution instead of organic solvents and proposed an organic-solvent-free AA-LPME method to extract and preconcentrate phthalic acid residues from edible oil samples. They combined this technology with liquid chromatography (LC)-diode array detector (DAD) to detect three types of phthalic acids (o-phthalic acid, m-phthalic acid, and p-phthalic acid) in edible oil samples, and the method showed low limits of detection (LODs) of 0.11–0.29 ng/mL and high extraction recoveries of 81–97%. Moreover, they compared this method with other dispersive methods, including ultrasound-assisted dispersive liquid–liquid microextraction (USA-DLLME), manual shaking-liquid-phase microextraction (MSh-LPME), and vortex-assisted liquid–liquid microextraction (VA-LLME). Under the same conditions, the amounts of time required by USA-DLLME, MSh-LPME, and VA-LLME were 12, 5, and 4 min, respectively, with the consumption of 5 mL of oil samples and 34 µL of the ammoniacal buffer. At the same time, the LODs for all the analytes were improved when using the proposed method compared with those of other methods [32]. This demonstrates that the organic-solvent-free AA-LPME method proposed above has the advantages of being more rapid, efficient, and sensitive. Most importantly, the application of an aqueous extractive phase instead of organic extraction solvents makes traditional LLE technology more environmentally friendly. Given that phthalic acid is the main hydrolysate of PAEs, it will be a good choice to adopt the method proposed above to indirectly determine the contents of PAEs in edible oils.

**Figure 2:**
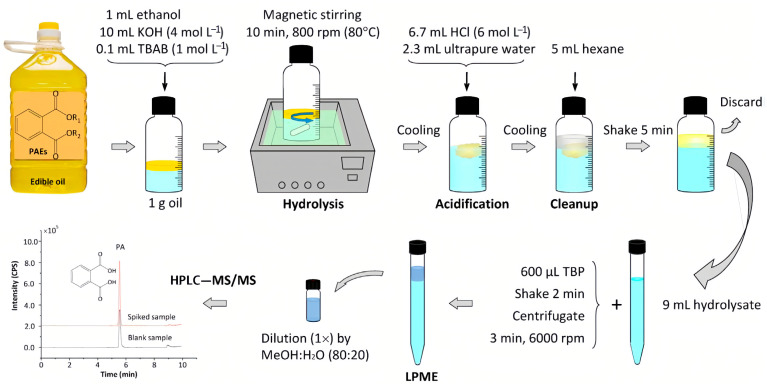
Schematic phase-transfer catalyst-assisted hydrolysis-LPME-LC-MS/MS procedure [31].

In addition, there are various PAE plasticizers in edible oils, some of which are still unknown due to the lack of corresponding standard compounds. Therefore, establishing a suitable method to determine all of the PAEs in edible oils is challenging. Liu et al. [31] and Xie et al. [41] used phase transfer catalyst to accelerate the oil/water biphasic base hydrolysis of PAEs; PAEs were hydrolyzed into phthalic acid, and the total contents of PAEs were indirectly measured by determining phthalic acid (Figure 2). This method provided new ideas for measuring the total contents of PAEs in edible oils.

### 2.2. Gel Permeation Chromatography

GPC uses a porous gel as the stationary phase. Based on the spatial size effect of the gel pores, molecules of different sizes elute from large to small in order, thus achieving the goal of separating the target analyte. GPC has the characteristics of high purification efficiency, reusability, wide applicability, and a high degree of automation. GPC is suitable for separating substances with significant differences in molecular size. The matrix of edible oil samples is complex and rich in a large amount of macromolecular interfering substances. Therefore, using GPC to remove macromolecular substances to purify small-molecule PAEs is a good method. In the pretreatment process of edible oil samples, macromolecular substances such as oils and pigments are first leached out, while small-molecule substances such as PAEs are leached out later, thus achieving effective purification.

Li et al. used GPC to extract 15 kinds of PAEs from edible oils with a glass chromatography column filled with a Bio-Beads (S-X3) filler. Then, the contents of the extracted solution were detected by GC-mass spectrometry (MS). The experimental results showed that the LODs of the 15 kinds of PAEs were between 0.001 and 2.000 µg/L, the average standard addition recoveries of three concentrations were in the range of 70.50–112.00%, and the average deviations (*n* = 6) were in the range of 1.59–7.54%. This indicated that GPC had a good purification ability for oil samples [23]. The application of the fully automatic purification and concentration of the GPC system greatly simplified the extraction process, enabling unattended and reliable operation [42]. However, GPC also has many shortcomings. In 2018, Li et al. found that in sesame oil, PAEs had a significant overlap with the matrix, and the interference of the matrix after the GPC approach was difficult to compensate for [24]. Moreover, conventional GPC technology consumes large volumes of solvents and has high costs. The GPC system itself has a PAE background value, which makes the quantification of trace PAEs difficult to achieve [24]. Therefore, in recent years, for the determination of PAEs in edible oils, GPC technology has gradually been replaced by other technologies, such as solid-phase extraction (SPE) and LLE.

### 2.3. Sorptive-Based Extraction Techniques

SPE is by far one of the most common sample preparation methods for the determination of PAEs [43]. When a complex sample solution passes through the extraction column, the sorbent will selectively retain the target substance and some of the interferents through polar, hydrophobic, or ion exchange interactions. Next, the extraction column is washed to remove interferents adsorbed by the sorbent, followed by choosing another solvent to elute the target analyte, thus achieving the purpose of separating, purifying, and enriching the target compound [44,45]. Compared with traditional LLE method, SPE has the advantages of avoiding emulsification phenomena, being easy to automate, and achieving efficient purification [25,26,27]. There have been many new reports on the extraction of PAEs in edible oils using SPE combined with various measurement methods (Table 3).

The development of SPE technology mainly depends on the innovation of the sorbents. For the extraction and purification of PAEs in edible oils, traditional sorbents for SPE mainly include C18 [46], primary secondary amine (PSA) [43,47,48], and Florisil^®^ [49,50]. However, conventional SPE sorbents exhibit low adsorption and selectivity. Chen et al. invented novel molecularly imprinted polymers of PAEs by atom transfer radical polymerization to replace traditional SPE sorbents. They successfully detected 10 PAEs in edible oils using molecularly imprinted solid-phase extraction (MISPE) combined with GC and a flame ionization detector (FID). The LODs of this method were 0.10–0.25 μg/mL, and the recoveries of the spiked samples were 82.5–101.4%. The extraction effectiveness of MISPE for PAEs was compared with that of commercial SPE columns, and the results indicated that the performance of MISPE was better than those of C18-SPE, PSA-SPE, PAE-SPE, and silica SPE under optimized extraction conditions [53].

Solid-phase microextraction (SPME) pretreatment method was developed on the basis of SPE. It is a new sample pretreatment technology that integrates sampling, extraction, enrichment, and injection into one step [60]. In SPME, the sorbent is coated onto a matrix material such as quartz fibers to extract, enrich, and concentrate the target compounds. Once the extraction process is finished, the fibers undergo desorption using either thermal desorption or liquid desorption methods. Subsequently, the target compounds are detected [61]. The entire process is easy to perform, time-saving, and solvent-free [62]. In SPME, the performance of the coating materials is the most critical factor in improving the extraction efficiency [63]. Therefore, similar to SPE, the development of SPME technology also mainly depends on the innovation of the coating materials. To date, various coating materials have been developed for SPME to extract PAEs from edible oils, such as metal-organic framework deep eutectic solvent/molecularly imprinted polymers (MOF-DES/MIPs) [3], polydimethylsiloxane/divinylbenzene (PDMS/DVB) fiber and polyethylene glycol (PEG) fiber [58], materials institute lavoisier-88(Fe)/graphene oxide (MIL-88(Fe)/GO)-coated fibers [54], graphene/polyvinylchloride (G/PVC) nanocomposite [57], and divinylbenzene–carboxen–polydimethylsiloxane (DVB/CAR/PDMS) [56].

**Figure 3:**
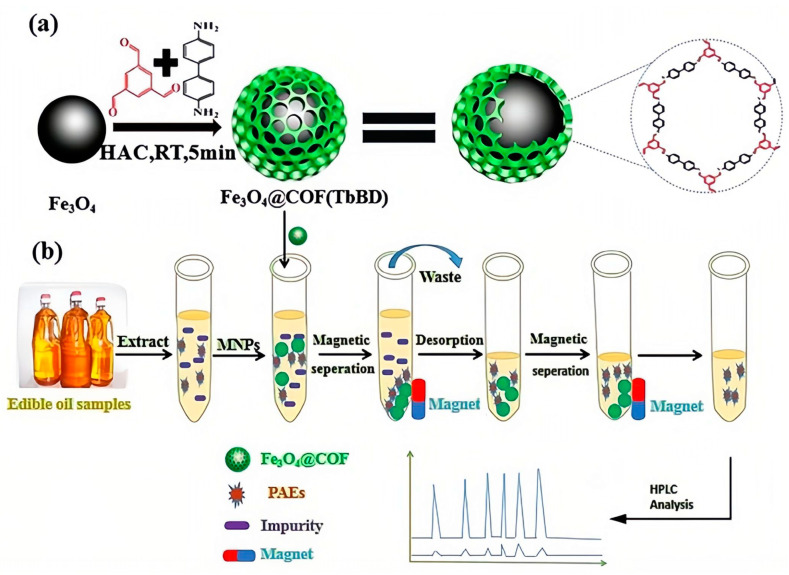
Schematic MSPE-LC-DAD procedure for the determination of PAEs in edible oils: (**a**) Fabrication process for Fe_3_O_4_@COF(TbBD) materials; (**b**) MSPE procedure for the detection of PAEs [4].

Magnetic solid-phase extraction (MSPE), which uses magnetic materials as sorbents and utilizes an external magnetic field to conveniently and quickly separate the adsorbent and analyte from the solution during the purification process, has also received great interest for achieving separation operation more easily [64]. Zhao et al. fabricated new magnetic covalent organic framework nanospheres named Fe_3_O_4_@covalent organic framework (1,3,5-triformylbenzene-benzidine) (Fe_3_O_4_@COF(TbBD)) as the magnetic sorbent and combined them with LC-DAD to achieve the extraction and detection of seven PAEs in edible vegetable oils (Figure 3). Under optimal conditions, the proposed method possessed a high sensitivity with LODs of 0.55–0.95 µg/kg and limits of quantification (LOQs) of 1.80–3.10 µg/kg, as well as satisfactory recoveries of 80.2–102.9% [4].

### 2.4. QuEChERS

**Figure 4:**
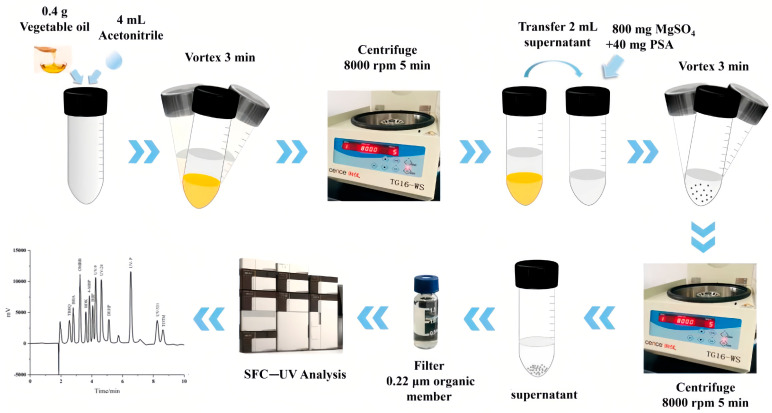
Schematic diagram of the QuEChERS-SFC method for the determination of PAEs in edible oils [29].

QuEChERS was first proposed by Anastassuades et al. in 2003 [28] and has received significant attention for its advantages such as its simplicity, low solvent consumption, and flexibility. In recent years, QuEChERS has gradually become a new trend for the detection of trace organic matter in foods, with a wide range of applications in the detection of plasticizers (Table 4). This method consists of two major steps: LLE with ACN (acetonitrile) and subsequent purification by dispersive solid-phase extraction (DSPE). As shown in Figure 4, Gan et al. successfully applied QuEChERS followed by supercritical fluid chromatography (SFC) and UV detector for the qualitative and quantitative analysis of 12 chemical additives (including three plasticizers: BBP, DEHP, and trioctyl trimellitate (TOTM)) in various edible vegetable oils [29]. In detail, they performed LLE with 0.4 g of edible vegetable oils and 4 mL of ACN, followed by salting-out with anhydrous magnesium sulfate. After DSPE with the sorbent, the supernatant was analyzed by SFC. In this process, they used ultracentrifugation to help with the purification.

In addition to SFC, QuEChERS is usually combined with other detection technologies, such as GC-MS/MS [30,65] and LC-DAD [66], to detect plasticizers in edible oils. All these methods have achieved satisfactory results. Although QuEChERS has been able to meet the extraction requirements for most of the tested samples, further removal of lipids from edible oils for more accurate detection of the target analytes is still of great significance. Sun et al. combined QuEChERS with freezing-lipid precision to further remove the lipids in the edible oil sample during the process of extracting PAEs, thus preventing the lipids from affecting the performance of the instrument [65]. The results showed that the extraction solution without freezing-lipid precipitation was turbid due to the dispersion of lipids in it, while the extraction solution after freezing-lipid precipitation was significantly clearer, and a large amount of lipids was deposited at the bottom of the glass centrifuge bottle. The effective removal of lipids would further improve the accuracy of detection and reduce the pollution of lipids on the instrument. As an emerging technology, QuEChERS will have an increasingly wider application in the pretreatment of edible oil samples.

## 3. Detection Techniques

At present, a variety of detection techniques have been developed to determine the contents of PAEs in edible oils, including GC, LC, immunoassay, SFC, and surface-enhanced Raman spectroscopy (SERS).

### 3.1. Gas Chromatography

GC is a common analytical technique with the advantages of high selectivity, fast analysis speed, less sample consumption, and less organic solvent consumption. A GC system usually consists of a carrier gas supply, injector, column, detector, data processing system, and temperature control system. Common detectors include thermal conductivity detectors (TCDs), FIDs, electron capture detectors (ECDs), flame photometric detectors (FPDs), and MS detectors. Of these, FIDs and MS detectors are often used to determine the contents of PAEs in edible oils. As a universal detector, the FID has the advantages of high sensitivity and low price. Khoshmaram et al. used a GC-FID system to detect PAEs in edible oils [33]. The LODs were 0.007–0.023 μg/L. However, the GC-FID has limited selectivity due to the complex matrix in the detection of PAEs in edible oils. Compared with the GC-FID method, the GC-MS method has a higher sensitivity and specificity. It is widely used in the detection of PAEs in edible oils [23,48,67,68]. When GC-MS is used for quantification, a quadrupole MS detector is mainly chosen. Wu et al. used GC combined with a quadrupole MS detector to quantitatively determine the contents of 17 PAEs in edible oils [48]. The LODs were in the range of 0.1–0.2 mg/kg. To reduce the recovery error caused by the complexity of the matrix and improve the accuracy, Oh et al. established an isotope dilution-GC-MS method for the detection of 12 PAEs in edible oils [50]. The relative standard deviations (RSDs) were 0.92–10.6%, and the recoveries were 80.6–97.8%. GC-MS/MS is also able to reduce the interference of the complexity of the matrix on the detection [22,30,69]. Lu et al. determined the contents of 10 PAEs in food samples using GC-MS/MS [69]. The recoveries of 10 kinds of PAEs were 73.7–98.1%, and the RSDs were 1.7–10.2%.

When GC is used to detect PAEs in edible oils, the mobile phase and column type also affect the column efficiency and sensitivity. The commonly used column type is a capillary column with a film thickness of 0.25 mm. Helium is commonly used as the mobile phase. Some researchers choose cheaper nitrogen as the mobile phase. However, compared with helium, as the mobile phase, nitrogen has poorer sensitivity. Using the same detector and column with the same inner diameter and film thickness, the LODs were 0.007–0.023 µg/L with nitrogen [33] as the mobile phase and 0.1–0.2 mg/kg with helium as the mobile phase [48].

### 3.2. Liquid Chromatography

LC is an analytical method with high efficiency, good repeatability, accuracy, and stability. The sample dissolved in the mobile phase can be separated and detected qualitatively and quantitatively according to the size and strength of the interaction with the stationary phase. The detectors commonly used for the detection of PAEs in edible oils of LC include DADs, ultraviolet (UV) detectors, and MS detectors. Ibrahim et al. used on-line SPE-LC-DAD for the detection of PAEs in palm oils [52]. Li et al. used LC-MS/MS to detect 16 PAEs in simulants from plastic food contact materials [24]. However, the inevitable problem of using LC to detect PAEs in edible oils is the contamination of the PAEs. Compared with GC, LC has more contamination sources, including filters, pipes, and solvents for the mobile phase. Many scholars have made efforts to reduce the phthalate contamination of LC. Pardo-Mates et al. placed a suppression column between the pump and the injection valve to prevent contamination of the instrument background [20]. Vavrouš et al. reduced the influence of phthalate contamination by equipping the analytical system with a contamination trap, a 50 mm reversed-phase chromatographic column [51]. Compared with LC-UV or DAD, LC-MS/MS is more selective for determining the molecular weight information of the mixture, is more reliable for the quantification of PAE isomer mixtures, and has shorter analysis time. Furthermore, it can better achieve the separation of isomer mixtures [24]. Therefore, LC-MS/MS is advantageous when detecting mixed isomers of multiple PAEs in edible oils.

When it comes to the type of column of LC, the C18 column and the amino column are commonly selected for the detection of plasticizers in edible oils. However, due to the short life of the amino column, the C18 column is more popular [52]. In addition, ultra-high performance liquid chromatography (UPLC) column with smaller size of the fixed phase filler is also chosen. It improves the column efficiency and achieves satisfactory analysis results [19]. The choice of mobile phase is often considered in obtaining a better peak shape and separation effect. Formic acid and ammonium formate have been used in the mobile phase of LC for the detection of PAEs in edible oils. The addition of methanol can improve the detection sensitivity, and ammonium formate can also be added to adjust the peak shape [19].

### 3.3. Immunoassay

An immunoassay is a simple and rapid analytical technique with the advantages of good selectivity, high specificity, and low cost. In the immunoassay process, the analyte and the corresponding antibody are combined in a solution or gel to form insoluble antigen-antibody complex precipitation to realize the qualitative and quantitative detection of the analyte. Cui et al. used the immunofluorescence technique (IFT) to detect diisobutyl phthalate (DiBP) in edible oils [70]. The LOD was 5.82 ng/mL. Compared with GC and LC, immunoassays have the advantages of shorter times and lower costs, but this method has less sensitivity. Immunosensor techniques integrate traditional immunodetection and biosensing techniques, which greatly improves the sensitivity of the immunoassay technique. Wang et al. detected DBP in edible oils by using a fluorescence ratio immunosensor based on dual-emission carbon quantum dot-labelled aptamers with the LOD of 5.0 μg/L [43]. The contents of the DBP in soybean oil samples determined by the sensor method and GC were 102.2 ± 2.79 μg/L and 108.7 ± 3.05 μg/L, respectively. The results of the two methods were consistent (*p* > 0.05). However, the analytical data of this method were greatly affected by the operating temperature. The IFT and immunosensor technique above rely on the combination of an antigen and antibody, which are only suitable for immunogenic molecules, and the preparation process is cumbersome. By contrast, a molecularly imprinted biomimetic immunoassay can be used for non-immunogenic molecules, which has the advantages of a low cost and easy preparation. Wang et al. prepared bionic antibodies and used the molecularly imprinted biomimetic immunoassay method based on a quantum dot maker to detect PAEs in edible oils [47]. The LOD was 0.011 mg/L, and the sensitivity was 0.136 mg/L. The crossing reaction values of the two structural analogues were 4.75% and 6.89%. In addition, the immunoassay method is suitable for rapid on-site detection.

The immunoassays described above have good specificity, which also limits their application scope. However, for most immunoassays, only one PAE in edible oils is commonly detected due to its inherent characteristics [21,43,47]. He et al. developed a polyclonal-antibody-based immunochromatographic assay (ICA) as a preliminary screen for the presence of phthalic acid in edible oils [46]. Phthalic acid is the hydrolysate of PAEs. An ICA strip can quickly detect 3 μg/mL phthalic acid in 5 min, which overcomes the shortcoming of commonly used immunoassay that only one PAE can be detected per test.

### 3.4. Other Technologies

In addition to the analytical methods above, SFC has also been applied to the detection of PAEs in edible oils. SFC is a chromatographic method using supercritical fluid as the mobile phase. As a relatively new chromatographic technology, SFC is commonly used to deal with substances that cannot be analyzed and separated by GC and LC. It has the advantages of being fast and having high efficiency. Gan et al. combined QuEChERS and SFC to detect plasticizers in edible vegetable oils [29]. Less than 10 min was required to separate 12 additives. Compared with other analytical methods, this method consumes fewer organic solvents and is greener. In addition, SERS has been applied for the detection of plasticizers in edible oils recently. Raman spectroscopy is based on the scattering spectrum that is different from the incident light frequency to obtain information about the molecular vibrations and rotations, which is then used for the study of the molecular structure. Since the Raman signal is relatively weak, it is necessary to use the enhancement effect when conducting Raman spectroscopy studies. Wang et al. synthesized nano-silver sol as the synergist and a two-dimensional silver plate as the SERS substrate to enhance the Raman scattering [71]. After the PAE was hydrolyzed to potassium hydrogen phthalate (PHP), quantitative detection was carried out directly. The LOD was 10^−9^ mol/L. This technique avoids the complex sample pretreatment process, and the detection time is short. It is a simple, fast, and lossless analytical method for samples, but the reproducibility of this method is relatively poor.

## 4. Conclusions

The detection of PAEs in edible oils is of great significance for controlling the quality of edible oils and ensuring public health. Related sample preparation methods and detection techniques have attracted the attention of scholars in recent years. This article reviews the recent reports on the sample preparation methods and detection techniques for the determination of PAE plasticizers in edible oils and explains the principles, advantages, and disadvantages of these methods.

Currently, LLE and SPE are the most commonly used sample preparation methods for the detection of PAEs in edible oils. With the improvement of technology and the popularization of green concepts, miniaturized technology has gradually attracted attention, and some more advanced pretreatment methods for edible oil samples have emerged, such as LPME, DLLME, SPME, MSPE, and QuEChERS. Compared with the traditional technologies, these advanced methods provide the advantages of being greener, more rapid, cheaper, and more accurate while consuming lower amounts of organic solvents. More importantly, some extraction technologies can even achieve zero consumption of toxic organic solvents, such as AA-LPME with an extractive phase of alkaline aqueous solutions. This type of organic-solvent-free technology is more friendly for operators and the environment, and its use will become a new trend in the development of pretreatment technology for edible oil samples in the future.

In addition, detection techniques have been greatly developed. Among these techniques, GC combined with various detectors, especially FIDs, is commonly used for the detection of PAEs in edible oils. The combination of GC and MS is also favored because of its high sensitivity and selectivity. Compared with GC, LC has greater advantages in the simultaneous detection of multiple PAEs in edible oils, which can better separate the mixture of isomers and achieve high-throughput detection. However, LC is limited by its vulnerability to PAE contamination. Therefore, reducing the influence of pollution is still a difficult problem in the widespread application of LC for the detection of PAEs in edible oils. In recent years, to meet the needs of on-site detection and reduce the time and economic costs, the detection of PAEs in edible oils based on immune technology has developed rapidly. Immunofluorescence and immunosensor techniques have been developed and applied in the detection of PAEs in edible oils. Overall, it can be predicted that the analytical methods of PAEs in edible oils will be developed to be more convenient, faster, and more accurate. At the same time, we are also looking forward to the development of superior analytical technologies to ensure the safety of edible oils for people.

## Figures and Tables

**Table 1:** Chemical and physical information of the seven commonly used PAEs.

Name	Abbreviation	CAS Number	Structure	Formula	Molecular Mass (g/mol)	Log P *
dimethyl phthalate	DMP	131-11-3	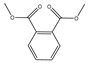	C_10_H_10_O_4_	194.18	1.98
diethyl phthalate	DEP	84-66-2	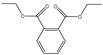	C_12_H_14_O_4_	222.24	2.69
dibutyl phthalate	DBP	84-74-2	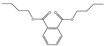	C_16_H_22_O_4_	278.34	4.63
benzyl butyl phthalate	BBP	85-68-7	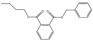	C_19_H_20_O_4_	312.36	5.03
di(2-ethylhexyl) phthalate	DEHP	117-81-7	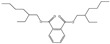	C_24_H_38_O_4_	390.56	8.03
dicyclohexyl phthalate	DCHP	84-61-7	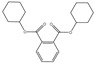	C_20_H_26_O_4_	330.42	5.58
diisononyl phthalate	DINP	28553-12-0	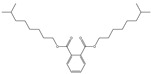	C_26_H_42_O_4_	418.62	8.76

* Log P values are obtained from ChemAxon. Log P is oil-water partition coefficient. Log P is the oil-water partition coefficient, which represents the logarithm of the ratio of the substance’s partition coefficient between n-octanol and water.

**Table 2:** Applications of LLE-based methods for extracting PAEs in edible oil samples.

Sample Amount	Analytes	Solvent Usage	Sample Preparation	Detection	LODs	Ref.
0.1 g	12 PAEs (DMP etc.)	Extraction solvent: 900 µL of n-hexane saturated acetonitrile	LLE	LC-HRMS	0.02–0.35 mg/kg	[19]
0.5 g	17 PAEs (DMP etc.)	Extraction solvent: 40 mL of hexane-saturated acetonitrile	LLE	GC-MS/MS	0.1–4.0 µg/kg	[22]
2 g	10 PAEs (DMP etc.)	Extraction solvent: 5 mL of acetonitrile	LLE	LC-MS/MS	1–9 µg/L	[20]
50 g	DEP	Extraction solvent: 75 mL of a mixture of hexane and acetone (1:1, *v*/*v*)	LLE	GNP-rt-IPCR	1.06 pg/L	[21]
1 g	PA (hydrolysate of total PAEs)	Extraction solvent: 600 µL of TBP	LPME	LC-MS/MS	1.0 µmol/kg	[31]
5 mL	o-PA, m-PA, p-PA	Extraction solvent: 34 µL of ammoniacal buffer	AA-LPME	LC-DAD	0.11–0.29 ng/mL	[32]
0.5 mL	5 PAEs (DMP etc.)	For AALLE, extraction solvent: 1 mL of DMSO;For DLLME, dispersive solvent: DMSO (from AALLE); extraction solvent: 0.45 mL of chloroform	AALLE and DLLME	GC-FID/GC-MS	0.007–0.023 µg/L	[33]

AA-LPME: air-assisted liquid-phase microextraction; DAD: diode array detector; DLLME: dispersed liquid-liquid microextraction; DMP: dimethyl phthalate; DMSO: dimethyl sulfoxide; DEP: diethyl phthalate; FID: flame ionization detector; GC: gas chromatography; GNP-rt-IPCR: gold nanoparticles improved real-time immuno-Polymerase Chain Reaction; HRMS: high-resolution mass spectrometry; MS: mass spectrum; PA: phthalic acid; TBP: Tributyl phosphate.

**Table 3:** Applications of SPE-based methods for extracting PAEs in edible oil samples.

Sample Amount	Analytes	Sorbents	Sample Preparation	Detection	LODs	Ref.
2 g	3 PAEs (DMP etc.)	C18	SPE	ICA	3 μg/mL	[46]
1 mL	DBP	PSA	SPE	Fluorescence ratio immunosensor method	5.0 μg/L	[43]
50 mL	DBP	ProElut PSA	SPE	MIBIA	0.011 mg/L	[47]
0.4 g	17 PAEs (DMP etc.)	Silica/PSA-mixed	SPE	GC-MS	0.1–0.2 mg/kg	[48]
0.5 g	16 PAEs (DMP etc.)	Florisil^®^	SPE	LC-MS/MS	31.9–390.8 µg/kg	[49]
4 g	3 PAEs (DBP etc.)	Florisil^®^	LLE and SPE	ID-GC-MS	4.6–10.0 µg/kg	[50]
2 mL	6 PAEs (DEP etc.)	C18-modified silica	LLE and SPE	LC-MS/MS	/	[51]
1 mL	6 PAEs (DMP etc.)	C16	Online SPE	LC-DAD	3 µg/L	[52]
2 g	10 PAEs (DMP etc.)	MIPs	MISPE	GC-FID	0.10–0.25 µg/mL	[53]
0.5 g	7 PAEs (DMP etc.)	MIL-88(Fe)/GO	SPME	GC-FID	0.5–2.0 ng/g	[54]
0.5 g	10 PAEs (DMP etc.)	PDMS	DI-SPME and LLE	GC-MS/MS	/	[55]
1 g	16 PAEs (DMP etc.)	DVB/CAR/PDMS	HS-SPME	GC-MS/MS	0.02–0.05 mg/kg	[56]
1 g	3 PAEs (DPP etc.)	G/PVC nanocomposite	HS-SPME	GC-FID	0.06–0.08 µg/L	[57]
5 mL	4 PAEs (DMP etc.)	PDMS/DVB	HS-SPME	GC-FID	2.6–3.3 ng/mL	[58]
/	4 PAEs (DMP etc.)	MOF-DES/MIPs	HFLMP-SPME	GC-FID	0.008–0.03 µg/L	[3]
1 g	7 PAEs (BBP etc.)	Fe_3_O_4_@COF (TbBD)	MSPE	LC-DAD	0.55–0.95 µg/kg	[4]
5 mL	5 PAEs (DMP etc.)	Co-MNPC@MIPs	MMISPE	GC-FID	0.010–0.025 μg/mL	[59]

BBP: benzyl butyl phthalate; CAR: carboxen; COF: covalent organic framework; Co-MNPC: cobalt-magnetic nanoporous carbon; DBP: dibutyl phthalate; DPP: di-n-propyl phthalate; DVB: divinylbenzene; G/PVC nanocomposite: graphene/polyvinylchloride nanocomposite; HFLMP-SPME: hollow fiber liquid membrane-protected solid-phase microextraction; HS-SPME: headspace solid-phase microextraction; ICA: immunochromatographic assay; ID: isotope dilution; MIBIA: molecularly imprinted biomimetic immunoassay; MIL-88(Fe)/GO: materials institute lavoisier-88(Fe)/graphene oxide; MIPs: molecularly imprinted polymers; MMISPE: magnetic molecular imprinting solid-phase extraction; MOF-DES/MIPs: metal-organic framework deep eutectic solvents/molecularly imprinted polymers; MSPE: magnetic solid-phase extraction; PSA: primary secondary amine; PDMS: polydimethylsiloxane; silica/PSA: silica/N-(nPropyl) ethylenediamine-mixed.

**Table 4:** Applications of QuEChERS-based methods for extracting PAEs in edible oils.

Sample Amount	Analytes	Sorbents (DSPE)	Sample Preparation	Detection	LODs	Ref.
2 g	6 PAEs (DMP etc.)	Z-Sep+ and C18	QuEChERS	GC-MS	0.02 mg/kg	[65]
0.5 g	15 PAEs (DMP etc.)	GCB and PSA	QuEChERS	GC-MS/MS	0.02–8.00 μg/kg	[30]
0.5 g	4 PAEs (DEP etc.)	primary secondary amine	QuEChERS and IL-DLLME	LC-DAD	6–9 ng/g	[66]
0.4 g	BBP; DEHP	PSA	QuEChERS	SFC-UV	BBP: 0.09 µg/mLDEHP: 0.12 µg/mL	[29]

DEHP: di(2-ethylhexyl) phthalate; GCB: gel permeation chromatography; IL-DLLME: ionic liq-uids-dispersive liquid-liquid microextraction; QuEChERS: quick, easy, cheap, effective, rugged, and safe; SFC: supercritical fluid chromatography.

## Data Availability

Not applicable.
